# Editorial: Harnessing nanotechnology for cancer treatment

**DOI:** 10.3389/fbioe.2025.1651124

**Published:** 2025-07-07

**Authors:** Merlis P. Alvarez-Berrios, Chuang Liu, Jingjing Sun

**Affiliations:** ^1^ Department of Natural, Computational and Exact Sciences, Inter American University of Puerto Rico at Ponce, Ponce, PR, United States; ^2^ Department of Neurosurgery, Brigham and Women’s Hospital, Harvard Medical School, Boston, MA, United States; ^3^ Department of Pharmaceutical Sciences, College of Pharmacy, University of Nebraska Medical Center, Omaha, NE, United States

**Keywords:** cancer, nanotechnology, bevacizumab, targeted drug delivery systems, theranostics

Cancer is the second leading cause of death worldwide. According to the American Cancer Society, it is projected that more than 35 million new cancer cases will occur by 2050 (American Cancer Society, 2024). Chemotherapy and radiation therapy are the most common treatments for this deadly disease. However, they damage both healthy and cancer cells, leading to severe side effects (Anand et al., 2023; Majeed and Gupta, 2023). In the last years, nanotechnology has gained significant interest as a promising tool to overcome these challenges. Therefore, this Research Topic collects comprehensive reviews focusing on nanotechnological approaches for cancer treatment.

Zhu et al. reviewed innovative applications of nanotechnology to address the limitations and challenges of conventional cancer treatments (Zhu et al.). Nanocarriers designed to selectively target cancer cells and respond to specific environmental signals within the tumor microenvironment can achieve effective delivery of therapeutic agents to the tumor cells. This approach not only enhances the efficacy of therapies by ensuring a higher concentration of the anti-cancer drug inside the tumor, but also reduces their toxicity to healthy tissues. Nanotechnology-based diagnostic technologies also show great potential to improve the effectiveness of treatments and survival rates. Nanoparticles, quantum dots, and other nanomaterials can be engineered to precisely interact with cancer biomarkers or tumor cells, allowing for early detection and treatment monitoring. The combination of diagnostic and therapeutic capabilities in a single nanoparticle has led to the development of theranostic nanoplatforms, which may play a crucial role in personalized cancer care in the future. The authors highlighted the use of gold nanoparticles, magnetic nanoparticles, and silica-based nanoparticles for this application. Moreover, the review also discussed the integration of nanotechnology with other therapeutic modalities such as immunotherapy, radiotherapy, and gene therapy. The challenges in translating these nanotechnologies to clinical practice, including the complexity of nanoparticle systems, their interaction with biological environments, issues related to biodistribution and toxicity, and the regulatory frameworks that govern the approval and use of nanomedicines were also addressed.

In a review by Zhan et al., the authors provided a comprehensive analysis of bevacizumab in ovarian cancer therapy with a focus on novel combination strategies, challenges, and future directions for enhancing bevacizumab-based regimens. Ovarian cancer is one of the deadliest malignancies of the female reproductive system. Its effective treatment remains a major challenge due to the limited sensitivity of screening methods and anti-cancer drug resistance (Zhan et al.). Bevacizumab, an anti-angiogenic therapy, has demonstrated promising results in clinical trials for ovarian cancer. However, it induces severe side effects such as hypertension, proteinuria, and gastrointestinal perforation. Moreover, cancer cells can develop resistance to bevacizumab, limiting its long-term efficacy. This review highlights the use of nanotechnology-based bevacizumab delivery systems to enhance the targeting specificity and safety of ovarian cancer treatment. The authors strongly recommended evaluating the long-term safety of these novel drug delivery systems and exploring additional nanotechnology applications for bevacizumab.

Among all cancer types, colorectal cancer is considered one of the leading causes of cancer-related mortality worldwide. Oral drug delivery systems have attracted considerable attention in the treatment of colorectal cancer due to their potential for targeted drug delivery and reduced side effects. Nevertheless, these systems encounter significant hurdles such as the highly acidic gastric environment (pH 1.0–3.0), enzymatic degradation by gastrointestinal digestive enzymes, and the barrier properties of the gastrointestinal mucosa, all of which significantly reduce drug bioavailability (Chai et al.). To address these challenges, Chai et al. provided a review article examining the use of oral nano-drug delivery systems as a promising strategy to significantly improve drug bioavailability and therapeutic precision. The review highlights the advancements in nano-drug delivery systems, focusing on the optimization of oral drug delivery systems, the development of tumor-specific targeting strategies, and the design of intelligent delivery systems responsive to the tumor microenvironment. The authors concluded that these nanotechnologies could significantly enhance the quality of life of colorectal cancer patients by leading to improved therapeutic outcomes and higher survival rates.

This Research Topic is further supported by a bibliometric study of nanotechnologies applied to hepatocellular carcinoma, conducted by Tuergank et al. using VOSviewer and CiteSpace. Trends in the literature indicate the key role of nanotechnology in advancing hepatocellular carcinoma diagnosis and treatment, with a focus on drug delivery and apoptosis.

In summary, nanotechnology provides a plethora of applications for effective cancer treatment, addressing many of the limitations associated with traditional therapies. It has the potential to revolutionize personalized cancer care by enabling precise targeting, early detection, and innovative therapeutic approaches.
